# Socio-cognitive correlates of intention to use Toombak: a cross-sectional study among students (13–16 years) in Khartoum State, Sudan

**DOI:** 10.1186/s12889-017-4606-z

**Published:** 2017-08-02

**Authors:** Hatim Mohammed Almahdi, Rouf Wahab Ali, Elwalid Fadul Nasir, Anne Nordrehaug Åstrøm

**Affiliations:** 1 Faculty of Dentistry, University of Science and Technology, Omdurman, Sudan; 2 Centre for International Health, University of Bergen, Bergen, Norway; 3 Department of Clinical Dentistry, Faculty of Medicine and Odontology, University of Bergen, Bergen, Norway; 4 Centre for oral health expertise, Hordaland-West, Bergen, Norway

**Keywords:** Toombak, Smokeless tobacco, Social cognitive, Social image, Social normative influence, Intention to use tobacco, Ever tobacco use, Sudan

## Abstract

**Background:**

Toombak is a form of smokeless tobacco, locally made and consumed in Sudan. It is associated with a number of health hazards, particularly oral cancer. This study was set out to assess the prevalence and socio-demographic distribution of its use, and to explore the socio-cognitive factors affecting the intention to use Toombak among secondary school students in Khartoum State, Sudan.

**Methods:**

A cross-sectional school-based study was conducted in 2013–2014 where schools were randomly selected using a one-stage stratified sampling procedure. The sample size was calculated to consist of 1526 students. Data were collected through a self-administered questionnaire, which contained some cognitive constructs; information received, social image, attitudes, normative social influence, accessibility to Toombak, socio-demographics and Toombak use related variables.

**Results:**

A total of 1670 secondary school students participated in the study. More than half of them 53.4% were <15-years-old and 53.6% were females. Only 5.3% of the students reported intention to use Toombak. Among the students 10.9% were ever Toombak users, 81.6% reported a positive attitude towards its use and 60.7% had received information about its harmful effects. A total of 72.6% reported normative social influence towards using Toombak and 62.5% perceived a negative social image attached to its use. Most of the students, 70.8% reported exposure to anti-Toombak information, 41.8% confirmed exposure to Toombak advertisement and 87.5% reported indirect access to its sale. Younger students reported ever use of Toombak less frequently than their older counterparts (38.4% versus 61.6%), *p* < 0.001. Males reported ever use of Toombak more frequently than did females (74.3% versus 25.7%), *p* < 0.001. According to the regression model, individuals who perceived a positive social image of Toombak users and had past experience were more likely to intend to its use.

**Conclusions:**

The present study suggested that the prevalence of Toombak use among Sudanese secondary school students is low and that male and older students are more frequent users. Students’ decision to use Toombak was based upon past experience with Toombak use and the social image attached to its use. Easy access to Toombak and encouragement from friends and classmates were among the factors which support intention to use Toombak but only in the unadjusted analyses.

## Background

Use of smokeless tobacco (SLT) is a health problem that affects more than 300 million people globally [1]. At least 1 in 10 adolescents (aged 13 to 15 years) use a form of tobacco. This number is even higher in some parts of the world [2]. SLT is more popular among adolescents of both sexes in some low- and medium-income countries (LMIC) in South-East Asia and Africa, than in high-income countries in Europe and the United States [3]. The Global Youth Tobacco Survey (GYTS) in Sudan has revealed that 10.2% reported use of other forms of tobacco than cigarettes (including Toombak and shisha) in 2005, This prevalence dropped slightly to 7.4% in 2009 and 4.9% in 2014, 6.1% boys and 3.2% girls reported currently use of SLT [4].

Toombak is a form of SLT that is locally made in Sudan and is associated with a number of health hazards, including oral cancer [5–8].

Adolescence (aged 10–19 years) [9], represents the transition from childhood to adulthood and is the period of initiation of use of any tobacco [10]. Tobacco consumption during this period can lead to physical and mental harm as well as addiction [11], making adolescents’ use of tobacco a significant public health problem.

Perceived accessibility and promotion of SLT increase the risk of tobacco initiation among adolescents. The World Health Organization (WHO) estimates that one-third of adolescents’ experience with using tobacco occurs as a result of exposure to advertisement and promotion [12, 13]. In addition, exposure to anti-tobacco information has the potential to reach a wider audience and educate both current and potential users. However, the efficacy in terms of health awareness of tobacco is questionable [14, 15]. Cultural acceptance and social image of the tobacco users may indeed play an important role in SLT use, as well as normative social influences [16, 17].

In order to understand and explain health-related behaviours such as the use of SLT, social cognition models have been developed and adopted in behavioural science research [18–20]. According to the socio-cognition models, knowledge about the health related hazards of a particular behaviour and attitudes towards that behaviour, together with social norms, are important cognitive factors influencing adolescents’ intention to perform a particular health-related behaviour [19]. Identification of socio-cognitive predictors of intention to use Toombak is a first step to explain that behaviour and thus to obtain necessary information for planning and implementing Toombak interventions. Therefore, the more one knows about a particular behaviour – the easier it becomes to change it [19].

The exact prevalence of Toombak use which is the principal SLT in Sudan needs specific emphasis. However, so far there is insufficient information neither about the cognitive factors predicting intentions to use Toombak nor the trends of its actual use among the younger Sudanese population [21]. Such information can contribute to improving primary and secondary tobacco preventive programs at both the individual and population levels. Focusing on secondary school students in Khartoum, Sudan, this study sets out to assess the prevalence and socio-demographic distribution of ever use of Toombak and to explore the socio-cognitive factors affecting intention to use it.

## Methods

A cross-sectional school-based study was carried out during 2013–2014 as a part of a larger research project focusing on the use of Toombak among secondary school students in Khartoum State, Sudan. Khartoum is the capital of the Sudan and consists of three cities (Khartoum, Omdurman, and Khartoum North), including seven localities; (Khartoum, Jabal Awaliya, Omdurman, Umbadda, Karary, Bahry and Sharg Alnil). The educational system comprises private and public schools as well as separate schools for boys and girls.

## Sampling procedures

The seven localities in Khartoum state include more than 643 secondary schools with a total number of about 200.000 students. For the purpose of this study, secondary schools in Khartoum State, the primary sampling unit, were randomly selected from a sampling frame consisting of 643 schools using a one -stage stratified (private/public, male/female) sampling procedure. A total of 28 schools, four from each locality (public/female, public/male, private/female and private/male), were randomly selected, with the substitution of the schools that failed to fulfil the inclusion criteria (acceptance to participate and ethical approval letter). All seven localities of the three cities are represented in the sample. It had been estimated that four schools in each locality would provide a satisfactory sample size calculated prior to the initiation of the study. Sample size calculation was made using the following equation: (*N* = 4 ${\mathrm{z}}_{\propto}^2$ P (1-P)/w2), Where N is the total population (200.000 students). Where P is the expected proportion (0.05) that have the characteristic of interest, W is the width of the confidence interval (equal to twice the “margin of error”), and zα is a value from the normal distribution related to and representing the confidence level (equal to 1.96 for 95% confidence). Then the following equation (*n*= n_0_/1+ n_0_/N) was used to determine the actual sample size which was found to be 1526 secondary school students. All students (census) in the age range (13–16 years) who attended the 28 selected schools were eligible to participate in the study. Eligibility criteria required the presence at the school at the time of the study and the provision by the student of an informed consent. A total of 1670 students were recruited, representing an additional 9% to the calculated sample size to account for incomplete filled questioaire.

### Data collection

The survey instrument was adopted from the Global Youth Tobacco Survey (GYTS); a school-based survey designed to collect information from the students in regard to the use of Toombak and its socio-cognitive and socio-demographic antecedents. GYTS is an integral part of the Global Tobacco Surveillance System (GTSS), constituting questions about smokeless tobacco, started by the WHO in 1999 [22].

A self-administered questionnaire was completed in classroom settings in the absence of the teachers but under the supervision of trained data collectors. The data collectors gave standardized instructions about the purpose of the study and completion of the questionnaire. Written informed consent was requested from participating students and their parents through the schools’ administration. Ethical clearance and approval were sought from the Ethical Committee of Faculty of Dentistry, University of Science and Technology, Ministry of Health and Ministry of Education in Khartoum state and also from Education authorities in each locality and school. The questionnaire was constructed in English and administered in Arabic. The questionnaire was translated from English into Arabic and subsequently back translated into English by experts in both languages. A pilot study testing the accuracy of translation and understanding of the questions was conducted before administration of the study in the selected schools. This pilot was conducted in two schools (male, female) including 60 students. Some minor adjustments of the survey instrument were performed before it was administered in the main survey.

### Questions and variables

#### Socio-demographic characteristics

*Age-group* was measured by *one question;* “what is your age”. *Parents’ employment* was measured by *one* question; “do your parents work”. *Parents’ education* was measured by *two questions*; “What level of education did your father complete”; “what level of education did your mother complete”.

*Exposure to anti-Toombak information* was assessed by *three questions*; “during the past 30 days, did you see any anti- Toombak advertisement in newspaper, TV, internet billboard”; “during the past 30 days did you see any advertisement about anti –Toombak information during social events, sports”. A sum variable “*exposure to anti-Toombak information”* was constructed from the three questions.

*Exposure to Toombak advertisement* was measured by *one question* “during the past 30 days did you see any Toombak promotion in the Toombak shops”.

*Received information about harmful effects of Toombak* use was measured by *four* questions; “has anyone in your family discussed the harmful effects of Toombak with you”; “during the past 12 months did you read in your school texts about the health effects of Toombak”; “during the past 12 month did you discuss in any of your classes the reasons why people at your age use Toombak”; “during the past 12 month did you receive information about side effects of Toombak use”. A sum variable “*Received information about harmful effects of Toombak* use” (Cronbach’s α 0.70).

*Attitudes towards Toombak use* were assessed by *four* questions; “are you in favour of banning Toombak in public closed places”; “once someone has started to use Toombak, do you think it would be difficult for him/her to quit”; “do you think Toombak use is harmful to your health”; “do you think Toombak use for only one or two years does not cause harmful effect”. A sum variable “*attitudes towards to Toombak use*” (Cronbach’s α = 0.54); was constructed from the four questions.

*Social image related to Toombak use* was assessed by *three* questions; “do you think Toombak helps people feel more comfortable or less comfortable at celebrations, parties, or in other social gatherings”; “do you think those who are using Toombak have more friends”; “do you think those who are using Toombak are more attractive”. A sum variable “*Social image* related *to Toombak use”* (Cronbach’s α = 0.54); was constructed from the three questions.

*Normative social influence* was assessed by *seven questions*; “does any one of your parents use Toombak”; “do any of your best friends use Toombak”; “how many of your class**-** mate use Toombak”; “how many days does anyone use Toombak in your presence inside your house”; “during the past 7 days, how many days any one used Toombak in your presence in closed public places”; “how many days any one use Toombak in your presence in open public places”; “during the past 7 days, how many days any one used Toombak (school personnel) in your presence inside the school buildings”. A sum variable “*normative social influence”* (Cronbach’s α = 0.68); was constructed from of the seven questions.

*Intention to use Toombak* was assessed by *two questions*; “if one of your best friends offered you Toombak, would you use it”; “at any time during the next 12 months do you think you will use Toombak”.

Ever users were defined as having used Toombak at least once or twice in their life [23]. Ever Toombak use was measured by *one question*; “have you ever tried or experimented the use of Toombak even once”.

*Access to Toombak* was measured by *one question*; “how did you get Toombak last time”.

### Statistical analyses

Data were analysed using the Statistical Package for the Social Science, version 20 (SPSS Inc. IL, USA). Descriptive analyses were performed using frequencies and percentages. Bivariate relationships between the dependent variable and each independent variable were assessed using cross-tabulation and Chi-square. Multiple variable analysis was conducted using stepwise logistic regression with the intention to use Toombak as the dependent variable. Independent variables that showed significant relationships with the intention to use Toombak in bivariate analysis were included in the multiple variable analysis. Estimates were presented as Odds Ratio (OR) and 95% confidence Interval (CI), in addition to Nagelkerke’s R2 to explain the variance of the model.

## Results

### Sample characteristics

A total of 1670 secondary school students present at school at the time of the survey participated in the present study, with response rate 100%. More than half of the students 53.4% (872) were <15-years-old and 53.6% (887) were females, 4.3% (70) of student’ parents were unemployed and 3.7% (45) had no education (Table 1).

**Table 1:** Frequencies (n) and percentages (%) of the socio-demographic characteristics of secondary school students in Khartoum State

Characteristics	%(n)
Age group	
< 15 years	53.4 (872)
≥ 15 years	46.6 (761)
Gender	
Female	53.6 (887)
Male	46.4 (768)
Parents’ Employment	
Unemployed	4.3 (70)
Employed	95.7 (1544)
Parents’ Education	
Not educated	3.7 (45)
Educated	96.3 (1170)

### Toombak related socio-cognitive variables

As shown in (Table 2) most of the students, 81.6% (1291) reported a positive attitude towards Toombak use and 60.7% (970) confirmed having received information about harmful effects of Toombak. Most of the students, 70.8% (1104), reported exposure to anti-Toombak information, and 41.8% (673) confirmed exposure to Toombak advertisement. The majority of the students 72.6% (1110) reported normative social influence towards using Toombak and 62.5% (1001) of them perceived a negative social image of Toombak users. Most of the students, 87.5% (232), reported indirect access to Toombak sales.

**Table 2:** Frequencies (n) and percentages of Toombak related variables among secondary school students in Khartoum State

Variables	% (n)
Attitude towards to Toombak use	
Negative attitude	18.4 (292)
Positive attitude	81.6 (1291)
Received information about Toombak	
No	39.3 (629)
Yes	60.7 (970)
Exposure to anti-Toombak information	
No	29.2 (455)
Yes	70.8 (1104)
Exposure to Toombak advertisement	
No	58.2 (937)
Yes	41.8 (673)
Normative social influence	
No	27.4 (418)
Yes	72.6 (1110)
Social image of Toombak user	
Negative	62.5 (1001)
Positive	37.5 (600)
Perceived Accessibility to Toombak	
Direct access	12.5 (33)
Indirect access	87.5 (232)
Intention to use Toombak	
No	94.7 (1532)
Yes	5.3 (86)
Toombak user	
No	89.1 (1465)
Yes	10.9 (180)

### Intended and ever Toombak use by socio-demographic variables

A total of 10.9% (180) with 95% CI (10.88, 10.91) reported ever use of Toombak. A total of 38.4% (68) students <15 years versus 61.6% (109) of those ≥15 years reported ever use of Toombak. Moreover, males reported ever use of Toombak more frequently than did females (74.3% versus 25.7%) *p* < 0.001, (Table 3).

**Table 3:** Frequency and distribution of ever Toombak users % (n) by socio-demographic variables

Variables	Ever Toombak use % (n)
Age group (years)	
< 15	38.4 (68)
≥ 15	61.6 (109)**
Gender	
Female	25.7 (46)
Male	74.3 (133)**
Parent education	
No	13.3 (6)
Yes	11.3 (132)
Parent employment	
No	2.9 (5)
Yes	97.1 (170)

**p < 0.001

A total of 5.3% (86) of the students with 95% CI (5.34, 5.36) intended to use Toombak. Bivariate analyses revealed that intention to use Toombak is statistically significantly associated with age (≥ 15 years) and male gender (*p* < 0.05), (Table 4).

**Table 4:** Frequency and distribution of intention to use Toombak %(n) by socio-demographic variables

Characteristics	Intention to use Toombak
Age group	
< 15 years	4.2 (36)
≥ 15 years	6.6 (49)*
Gender	
Female	3.5 (30)
Male	7.5 (56)**
Parent employment	
Not employed	4.4 (3)
Employed	5.3 (80)
Parent education	
Not educated	4.5 (2)
Educated	5.4 (62)

*p ≤ 0.05, **p ≤ 0.001

Intention to use Toombak by socio-cognitive variables and ever use of Toombak as depicted in (Table 5), bivariate unadjusted cross tabulation revealed that intention to use Toombak was statistically significantly associated with normative social influence (confirmed social influence 6.8% versus disconfirmed social influence 0.7%), with social image of Toombak use (positive image 8.7% versus negative image 3.2%, *p* < 0.001) and perceived accessibility to Toombak. Students who confirmed ever use were more likely than non-users to intend to use Toombak (22.3% versus 3.0%, *p* < 0.001).

**Table 5:** Frequency and distribution of intention to use Toombak % (n) by attitudes, Toombak information and exposure, social image, normative social influences and perceived accessibility

Variable	Intention to use Toombak % (n)
Attitude towards to Toombak use	
Negative	4.2 (12)
Positive	5.4 (68)
Received information about Toombak	
No	4.8 (10)
Yes	5.2 (68)
Exposure to anti-Toombak information	
No	4.7 (21)
Yes	5.4 (59)
Exposure to Toombak advertisement	
No	4.8 (44)
Yes	6 (40)
Normative social influence	
No	0.7 (7)
Yes	6.8 (74)**
Social image of Toombak user	
Negative	3.2 (32)
Positive	8.7 (51) **
Perceived Accessibility to Toombak	
Direct	31.2 (10)**
Indirect	11.1 (25)
Ever Toombak use	
Non-users	3 (43)
Ever user	22.3 (39)**

*p ≤ 0.05, **p ≤ 0.001

**Fig. 1:**
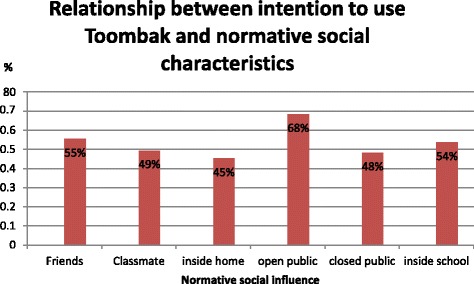
Frequency and percentage distribution of normative social influence factors and intention to use Toombak

Analysing social normative influence, the use of Toombak by friends 55.4% (46), (*p* ≤ 0.001), use of Toombak in open public 68.3% (56), (*p* ≤ 0.001), and use of Toombak inside school 53.6% (45), (*p* ≤ 0.001) significantly affect the likelihood of Toombak use intentions (Fig. 1).

Using multiple logistic regression analysis, intention to use Toombak was regressed on normative social influence, social images, perceived access to Toombak and ever use of Toombak, whilst adjusting for age and gender. Socio-demographic factors (age and gender) were entered in the first step; revealing a Nagelkerke’s R2 of 0.07, explaining 7% of the variance in Toombak use intentions. Normative social influence and social images were entered in the second step providing a Nagelkerke’s R2 of 0.16, thus raising the explained variance from 7% to 16%. Access to Toombak was entered in the third step increasing the Nagelkerke’s R2 from 16% to 0.19%. Ever use of Toombak were entered in the fourth step, the corresponding Nagelkerke’s R2 was 0.303. The final model explained 30.3% of the variance intention to use Toombak. Compared to females and ever users of Toombak, males and those who had never used Toombak were less likely to intend to use it. The corresponding ORs and 95% Confidence intervals (CI) were, 0.74 (0.25-0.2.20), 0.13 (0.04-0.39) respectively. Students who had a positive social image of Toombak user were more likely to intend to its use (OR 2.83; CI (1.18-6.83) (Table 6).

**Table 6:** Stepwise logistic regression of socio-demographic, normative social influence, social image, access to Toombak and ever Toombak use by intention to use Toombak among secondary school students in Khartoum State

Characteristics	Intention to use Toombak OR (95% CI)			
	Step 1^a^	Step 2^b^	Step 3^c^	Step 4^d^
Age group				
< 15 years	1	1	1	1
≥ 15 years	2.04 (0.91-4.60)	1.86 (0.80-4.28)	1.62 (0.69-3.83)	1.47 (0.59-3.63)
Gender				
Female	1	1	1	1
Male	2.45 (1.04–5.78)*	1.88 (0.75–4.68)	1.94 (0.76–4.91)	0.74 (0.25–0.2.20)
Social influence				
NO		1	1	1
Yes		2.64 (0.56–12.51)	2.19 (0.45–10.54)	2.63 (0.52–13.21)
Social image				
Negative image		1	1	1
Positive image		3.39 (1.47–7.76)**	3.16 (1.36–7.35)*	2.83 (1.18–6.83)*
Toombak access				
Direct access			1	1
Indirect access			0.32 (0.12–0.83)*	0.38 (0.14–1.05)
Ever Toombak use				
Ever user				1
Non-users				0.13 (0.04–0.39)**

^a^R2 =0.07, ^b^R2 = 0.16, ^c^ *R* = 0.19, ^d^R2 =0.30, *p ≤ 0.05, **p ≤ 0.001

## Discussion

This study is aimed to assess the prevalence and socio-demographic distribution of ever use of Toombak and to identify socio-cognitive predictors of intention to use it among secondary school students in Khartoum State, Sudan. The observed prevalence of ever use of Toombak was low, amounting to 10%, but varied systematically according to age and gender. The prevalence rate observed is fairly consistent with those obtained generally in other African countries amounting to less than 10%, e.g. as among Kenyan adolescents where prevalence rate was observed to be 9% [24]. The present findings are not comparable with SLT prevalence rates observed in India and Nepal amounting to 12.5% and 16.2% respectively [25, 26], and are also lower than those observed among Norwegian adolescents amounting to 11.9% [27].

The present study further revealed that the prevalence of ever use of Toombak as well as the prevalence of intention to use Toombak were higher among males than among females. A similar age and gender gradient in use of SLT have been confirmed among Norwegian adolescents [28]. The current finding that males have higher probability of being ever users than females were also found in previous studies from Sudan (9.5% males vs. 4.3% females), Nepal (13.2% males vs. 5.3% females) and Sri Lanka (12.4% males vs. 5.8% females) [29–32]. It is obvious that being a male is strongly associated with tobacco use [33]. One possible explanation may be that use of tobacco is believed to be associated with characteristics such as dominance, maturity and acceptance in the male community, thus reflecting the culture and also the effect of advertisement that link masculinity with the use of tobacco [34, 35].

The social gradient in health leading to health inequities is a worldwide phenomenon [36]. In this study, the social gradient was not observed regarding students’ intention to use Toombak and ever use of Toombak, as the frequency of Toombak use did not differ statistically significantly between students having and not having employed and/nor educated parents. In spite of a commonly observed association between socio-economic status (SES) and health behaviours, Hanson et al. [37] concluded that with respect to tobacco use this social gradient was not as strong among adolescents as among adults. In a study from India, Mathur et al. [38] demonstrated changes in the social gradient of tobacco users over the years, with adolescents from both low and high SES were at risk of becoming tobacco users.

Among the socio-cognitive factors investigated in this study, perceived social image and accessibility remained independent significant predictors of intention to use Toombak in the final step of the multiple variable logistic regression model. In the context of the present study, perceived social image was defined as adolescents’ opinion on how attractive, popular and trendy is a person who uses Toombak is, and this may reflect Ajzen’s [19] concepts of personal attitudes and subjective norms. Adolescents who were attaching a positive social image to Toombak use were more likely than their counterparts to report a stronger Toombak use intention. This is consistent with the findings of a study from Nigeria which suggests that social acceptance is recognized as the most common reason for SLT use [39]. Moreover, the relationship between the perceived social image and SLT use was found to be stronger than the association between the perceived social image and smoking, probably because SLT is recognized as a harm-reducing alternative to cigarettes and thus more socially acceptable [40, 41]. A study among Norwegian adolescents has shown that young males perceive SLT as trendy and socially attractive [28]. Although those adolescents intending to use Toombak were more likely to receive support from important other persons in unadjusted analyses, this relationship was not maintained in the final step of the multivariate logistic regression analysis, probably due to a confounding or mediation from other variables included in the model [17]. As shown in Fig. 1, beliefs about the opinion of important other persons, such as parents, friends and classmates played an important role in influencing adolescents’ intention to use Toombak. Similar findings have been reported from a study in Sudan as well as in studies from other parts of the world [29, 31, 42, 43]. A commonly reported influencing factor of adolescents use of tobacco products is peer’s pressure [44].

Previous studies have shown that perceived accessibility to tobacco increases the likelihood of tobacco use among adolescents [12, 45]. This relationship was also found in the present study, but only in unadjusted bivariate analyses.

A relationship between advertisement at the point of sale and intended use of Toombak was not observed in this study although a previous study from Sudan revealed that the promotion of tobacco products was a risk factor for actual tobacco use among adolescents [31]. The association between exposure to advertisement and tobacco use has been confirmed by Robertson et al. [46]. On other hand, Paynter et al. [47] reported limited evidence for the influence of point of sale marketing on tobacco use.

This study found that past behaviour in terms of ever use of Toombak was a strong predictor of Toombak use intention in the multiple variables analysis model and increased the predictive power of the model from about 19% to 30%. The direct effect of ever use of Toombak beyond the effect of socio-cognitive variables may be explained in several ways. First, it may be that users of Toombak do not make up a decision to use Toombak, but rather make up a judgment of what they are going to do based on past and recent performance. “I have used Toombak before and I will probably do so in the future”. Secondly, it may be that central socio-cognitive predictors of intended use of Toombak are not included in the predictor model. The residual effect of prior behaviour on intended and future behaviour in socio-cognition models have been extensively discussed by Ajzen in terms of the sufficiency of the socio-cognitive models [48]. Also, Ajzen suggests that when individuals have ambivalent or uncertain attitudes, the effect of prior behaviour will strongly influence intention. Accordingly, the prior behaviour is assumed to reflect all other factors that may influence intention and behaviours in the socio-cognition models [19]. Ouellette and Wood argued that “when a behaviour is performed routinely in a stable context, habitual responses are established, and accordingly, past-behaviour rather than intention will best predict future-behaviour” [49–51].

The most apparent limitation of this study is its’ cross-sectional design. This design makes it difficult to confirm any hypothesis and a cause-effect relationship between socio-cognitive factors and intention of Toombak use cannot be confirmed. As illustrated by the direct effect of past Toombak use, there are presumably several other factors not measured in this study that influence intention to use Toombak among adolescents in Sudan. In addition, the weak effect of normative social influence in this study may be attributed to lack of measurement of other normative variables (e.g. moral or descriptive norms) [52]. Self- reported intention and ever use of Toombak could have led to the potential recall and social desirability bias. Thus the results should be interpreted cautiously. Nonetheless, it portrays an important picture of the current situation.

## Conclusions

The study suggests that prevalence of Toombak use among Sudanese school students is low and that males and older students are more frequent users of Toombak than counterparts. Furthermore, students’ intention to use Toombak is based upon considerations regarding past experience with Toombak use and the social image attached to using it. Students would decide to use Toombak if they have previous experience and perceived a positive social image. Students’ intention is also influenced by perceived easy access to Toombak and having support from friends and classmates. Further research should incorporate a measure of observed use of Toombak to check the validity of self- reported use of Toombak employed in the present study.

The present findings could provide guidance for policy makers to develop tailored interventions regarding prevention of Toombak use among adolescents.

If the aim is to counteract the increase in Toombak consumption among adolescents, their intention to use Toombak should be addressed. If the use of Toombak is perceived to be socially attractive, popular and trendy among adolescents in Sudan, this is likely to contribute to a stronger intention to use Toombak and probably also towards actual use. Perceived positive social image which relates to Toombak users need to be addressed through extensive programs to change the culture of social acceptability of Toombak users into negative social image thus promoting a shift in social norms away from using Toombak among adolescents in the Sudan.

## Abbreviations

FCTC: Framework convention on tobacco control
GSPS: Global school personnel survey
GTTS: Global tobacco surveillance system
GYTS: Global youth tobacco survey
LMIC: Low and medium income countries
SLT: Smokeless tobacco
WHO: World Health Organization
